# Predictors of physical and mental health in persons with morbid obesity attending a patient education course – a two-year follow-up study

**DOI:** 10.1186/s12955-017-0675-z

**Published:** 2017-05-15

**Authors:** Anners Lerdal, Caryl L. Gay, Tore Bonsaksen, May Solveig Fagermoen

**Affiliations:** 1 Department of Nursing Science, University of Oslo, Faculty of Medicine, Institute of Health and Society, P.O. Box. 1130, Blindern, N-0318 Oslo, Norway; 2 Department for Patient Safety and Research, Lovisenberg Diakonale Hospital, P.O. Box 04970, Nydalen, N-0440 Oslo, Norway; 3 Department of Family Health Care Nursing, University of California at San Francisco, 2 Koret Way, San Francisco, CA 94143 USA; 4 Department of Occupational Therapy, Prosthetics and Orthotics, Oslo and Akershus University College of Applied Sciences, Faculty of Health Sciences, P.O. Box 4, St. Olavs Plass, N-0130 Oslo, Norway; 5 VID Specialized University, Faculty of Health Studies, Vågsgaten 40, 4306 Sandnes, Norway

**Keywords:** Obesity, Health-related quality of life, Personal factors, Coping, Self-esteem, Self-efficacy, Patient education

## Abstract

**Background:**

People with morbid obesity (body mass index ≥40) may experience changes in their health after participating in a tailored patient education course. The aims of this study were to assess the changes in physical and mental health in persons with morbid obesity during the 2 years following an educational course and to explore possible socio-demographic, treatment, and personal predictors of physical and mental health outcomes.

**Methods:**

In this prospective longitudinal cohort study, self-report questionnaire data were collected from people with morbid obesity at the beginning of mandatory educational courses while on a waiting list for gastric surgery and at two-year follow-up. Of the 185 who attended the courses, 142 (77%) volunteered to participate in the study, and the 59 with complete data at the two-year follow-up were included in the analysis. Physical and mental health were measured with the physical and mental component summary scores from the Short Form 12v2. Self-esteem was measured by the Rosenberg Self-Esteem Scale, and self-efficacy by the General Self-Efficacy Scale.

**Results:**

The participants reported better physical health at two-year follow-up than at baseline. Mental health did not change significantly over time. Receiving surgical treatment during the study period predicted better physical health at two-year follow-up, even after controlling for physical health at baseline. Mental health at baseline was the only significant baseline predictor of mental health at follow-up. However, increasing self-esteem and self-efficacy over the two-year study period independently predicted better mental health at follow up after controlling for mental health at baseline.

**Conclusion:**

Our study showed that people with morbid obesity on a waiting list for bariatric surgery improved their physical health during the 2 years after attending a tailored patient educational course. Improving self-esteem and self-efficacy may be important personal factors for maintaining mental health during this period.

**Trial Registration:**

NCT01336725. Registered 14 April 2011.

## Background

In most parts of the world and particularly in the USA, the proportion of people with morbid obesity (body mass index [BMI] of 40 kg/m^2^ or greater) is rapidly increasing [1]. In addition to reducing physical [2] and mental [2, 3] health-related quality of life (HRQoL), morbid obesity increases the risk for other diseases and health problems, such as diabetes, heart disease, musculoskeletal pain, obstructive sleep apnoea, hypertension, stroke, and cancer [4, 5].

In Norway, patients with morbid obesity awaiting surgical treatment may be offered a 40-h comprehensive patient education course at one of the country’s Patient Education Resource Centres. The course is taught by health care professionals in cooperation with previous course participants and includes information about available treatments, their possible complications and consequences for physical and mental health, and the importance of life style changes. The therapeutic approach is grounded in social-cognitive behaviour theory and focuses on identifying hidden resources, strengthening self-concept and social skills, and raising awareness of lifestyle choices. The educational course aims to help participants to make more informed decisions about whether they want surgery or alternative treatment and to achieve a healthier lifestyle and thereby improve their HRQoL.

In accordance with the International Classification of Functioning, Disability and Health (ICF), Wilson and Cleary [6] suggest that, in addition to peoples’ disease and body functions and structures, personal factors (defined as variations in individual beliefs, preferences, and values), and environmental characteristics are related to an individual’s overall quality of life. In a previous report from the baseline data of this study [7] evaluating selected socio-demographic factors (i.e., age, sex, level of education, marital status, living with children, work status, and social support) as well as personal factors (i.e., general self-efficacy, self-esteem, and sense of coherence), we showed that lower age, paid work, and high self-esteem were directly related to higher physical health. Furthermore, personal factors were directly related to people’s mental health and accounted for 41.6% of the variance in mental health. Self-efficacy in people with obesity has been shown to be influenced by their life companion [8], and has also been associated with their work status [9], highlighting the need to control for socio-demographic factors when evaluating the role of personal factors. A systematic review concluded that there is a lack of knowledge regarding the role of personal factors in relation to quality of life among obese individuals [10]. Previously, we have reported that having paid work and receiving social support from close friends and family predicted higher HRQoL 1 year after attending the educational course [9]. In our prior repeated measures analysis of change over time, we found that the participants had linear improvements in physical health, but no change in mental health during the first year after the course [11]. We found no studies examining which personal factors predict long-term physical and mental health outcomes among morbidly obese people attending an educational course. Moreover, treatment factors, such as whether or not the person had bariatric surgery following the educational course, are likely to affect long-term physical and mental health, and need to be considered when evaluating the role of personal factors.

Thus, the aims of this study were to assess the changes in physical and mental health in persons with morbid obesity during the 2 years following an educational course and to explore possible socio-demographic, treatment, and personal predictors of physical and mental health outcomes. We hypothesized that change in self-esteem and self-efficacy would predict better mental health but not better physical health at two-year follow-up.

## Methods

This article reports findings from a prospective longitudinal study of two cohorts, one with obesity and the other with chronic obstructive pulmonary disease (COPD), in which questionnaires were used to collect data over a two-year period. The methods are described in detail in a previous publication [7, 12].

### Sampling of participants

Participants were recruited at three different sites on the first or second day of 10 mandatory courses held in 2009. The 185 course participants were invited to participate in the study after receiving written and verbal information about it. A total of 142 participants enrolled in the study (77% response rate) and completed the questionnaire in a secluded room on-site. The participation rate was similar to that of prior studies [13, 14]. Their responses were returned in a sealed envelope and collected by the project representative. Follow-up questionnaires were mailed to the 142 study participants five times with the following response rates: 2 weeks (72.5%) and 3 (68.3%), 6 (55.6%), 12 (50.0%), and 24 (43.7%) months after completion of the course. Only data from baseline and the two-year follow-up are used in this study. Other findings have been previously reported [7, 9, 11, 12].

### Measurements

#### Physical and mental health

Physical and mental health were measured with the Short Form 12, version 2 (SF-12v2), a widely used abbreviated form of the SF-36 [15]. The 12 items assess eight dimensions of HRQoL [16, 17]. The raw scores on the eight dimensions are converted to scales from 0 (lowest QoL) to 100 (highest QoL). A physical component summary (PCS) and a mental component summary (MCS) score were computed [17] and served as the study’s primary outcomes. Since the main study compared HRQoL in two different cohorts, the generic SF-12v2 measure of HRQoL was selected.

#### Socio-demographic variables

Data for age (years), sex, marital status (married/cohabitant versus not married/not cohabitant), and employment status were collected. Participants’ level of formal education was categorized as having less than or equal to 12 years education or more than 12 years education.

#### Personal factors

The *Rosenberg Self-Esteem Scale* (RSES) [18] was used to assess participants’ global self-esteem. Rosenberg [19] proposes the attributes of a person with high self-esteem are: “self-respect, considers himself a person of worth”. The original RSES consists of ten statements with responses ranked from 1 ‘strongly agree’ to 4 ‘strongly disagree’. Our study used an abbreviated 4-item Norwegian version (RSES-4), which was developed using linear regression analysis and is highly correlated (*r* = 0.95) with the full 10-item version [20]. The sum-scores for participants can range from 4 to 16, with higher scores representing lower self-esteem. The Cronbach’s α of 0.84 for this study was similar to that of another Norwegian study [21] and indicates acceptable internal consistency reliability.

The *General Self-Efficacy Scale* (GSE) [22] measures optimistic self-beliefs in coping with the demands of life. It consists of 10 statements that respondents rate on a scale from 1 ‘completely disagree’ to 4 ‘completely agree’. The sum-score is calculated by summing each individual’s item scores. The sum-scores can range from 10 to 40, with higher scores indicating higher self-efficacy. High correlations with self-appraisal, self-acceptance, and optimism indicate theoretical accuracy of the self-efficacy concept [23], and factor analysis of the GSE has consistently produced the one-factor solution as used in this study. Item-total correlations range between 0.25 and 0.63, with factor loadings ranging between 0.32 and 0.74, and Cronbach α = 0.82 [24]. In the present sample, the GSE’s Cronbach α = 0.92, which indicates excellent internal consistency [25].

### Statistical analysis

Data were analysed using SPSS for Windows version 24 (IBM Corp, Armonk, NY). *T-*tests were used to compare groups on continuous variables. Group comparisons of ordinal and categorical data were conducted using chi-square tests. Pearson’s correlation coefficient (r) was used for correlation analysis. In order to compare participants’ scores from the different HRQoL domains, the scales were standardized based on a survey of the general US population. A score of 50 corresponded to the mean score and a deviation of 10 corresponded to one standard deviation in relation to the US-derived standard [16].

Three separate sets of hierarchical linear regression analyses were performed. The first set of regression analyses included the following independent variables as assessed at baseline: work status, self-esteem, and self-efficacy (Table 2). The dependent variables for the first set of regression models were baseline levels of physical and mental health, respectively. In the second set of regression analyses (Table 3), baseline work status, self-esteem, and self-efficacy were used to predict physical and mental health at the two-year follow-up. In the second step of this analysis, surgical treatment during the first year after the course was added to the model. The baseline level of the dependent variable was also included as an independent variable in the third step. The third set of regression analyses (Table 4) assessed whether baseline work status, changes in self-esteem, and changes in self-efficacy during the follow-up period were associated with changes in physical and mental health. As in the previous analysis, surgical treatment and baseline levels of the dependent variable were included as independent variables in the second and third step, respectively. Change scores were calculated as the difference between baseline scores and the scores at two-year follow-up.

Because all bivariate correlations between variables used in the analysis were *r* < 0.7, we assumed no multi-collinearity of variables. Cronbach’s α was used to assess the internal consistency of the scales. Cohen’s *d* was used as a measure of effect size for group differences, with 0.1 indicating a small effect, 0.3 a medium effect, and 0.5 a large effect [26]. The level of significance was set at *p* < 0.05. All tests were two-tailed. A sample size of 58 was sufficient to detect medium/large effects (i.e., f^2^ > 0.25) using multivariate regression analysis with 5 predictors, assuming a significance level of 0.05 and 80% power.

## Results

### Study population and sample

Of the 185 individuals invited to participate in the study, 142 (76.8%) consented and completed the baseline assessment. At baseline, the mean age of the 142 study participants (*M* = 42.5 years, *SD* = 10.4) and 43 non-participants (*M* = 44.2, *SD* = 9.1) was not significantly different (*p* = 0.33). Similarly, the proportion of women was not significantly different between the 142 study participants (70.4%) and 43 non-participants (60.5%, *p* = 0.22). At the two-year follow-up assessment, 59 (41.5%) of the original 142 participants returned the completed questionnaires, a retention rate comparable with prior studies [13, 14]. These 59 persons constitute the sample included in this analysis.

### Attrition analysis

Due to participant dropout over the two-year period, the sample included in this analysis was compared with the participants who dropped out with regard to their age, sex, and baseline measures of physical and mental health and personal variables. The mean age of the 59 participants in the sample (*M* = 44.2, *SD* = 9.7) did not differ significantly from the 83 individuals who dropped out (*M* = 41.2, *SD* = 10.8, *p* = 0.09). There were also no differences in the proportion of women in the study sample (*n* = 43 of 59, 72.9%) compared with those who dropped out (*n* = 57 of 83, 68.7%, *p* = 0.59). Moreover, no significant differences between the study sample and the dropouts were found on baseline physical health scores (sample *M* = 32.8, *SD* = 12.5, dropouts *M* = 33.7, *SD* = 11.4, *p* = 0.68, *d* = 0.08), nor on baseline mental health scores (sample *M* = 46.2, *SD* = 11.5, dropouts *M* = 43.1, *SD* = 12.1, *p* = 0.14, *d* = 0.26). Similarly, no significant differences between participants and dropouts were found with regard to self-esteem scores (sample *M* = 9.3, *SD* = 2.7, dropouts *M* = 10.0, *SD* = 2.6, *p* = 0.11, *d* = 0.26) or self-efficacy scores (sample *M* = 26.9, *SD* = 6.2, dropouts *M* = 26.2, *SD* = 6.5, *p* = 0.57, *d* = 0.11).

### Demographic characteristics of the sample

The socio-demographic and clinical characteristics of the participants are presented in Table 1. No sex differences were found on any of the variables.

**Table 1:** Characteristics of the study sample at baseline (*N* = 59)

Characteristics	Scale	All	Men	Women		
		*N* = 59	*n* = 16	*n* = 43		
Socio-demographic variables		*M (SD)*	*M (SD)*	*M (SD)*	*t*	*p*
Age		44.2 (9.7)	46.3 (7.7)	43.5 (10.4)	0.98	0.33
		*n* (%)	*n* (%)	*n* (%)	χ^2^	*p*
Working		41 (69.5)	12 (75.0)	29 (67.4)	0.31	0.58
Education >12 years		22 (37.3)	5 (31.3)	17 (39.5)	0.34	0.56
Paired relationship		41 (69.5)	14 (87.5)	27 (62.8)	3.36	0.07
Personal variables		*M* (*SD*)	*M* (*SD*)	*M* (*SD*)	*t*	*p*
Self-esteem (RSES-4, high scores = low self-esteem)	4–16	9.3 (2.7)	9.1 (2.5)	9.4 (2.8)	−0.42	0.68
Self-efficacy (GSE, high scores = high self-efficacy)	10–40	26.9 (6.2)	26.6 (6.4)	27.0 (6.2)	−0.23	0.82
Clinical variables		*n* (%)	*n* (%)	*n* (%)	χ^2^	*p*
Performed surgery		31.3 (66.1)	11 (68.8)	44 (74.6)	0.39^a^	0.52
Health-related quality of life (high scores = high quality)		*M (SD)*	*M (SD)*	*M (SD)*	*t*	*p*
Physical health	0–100	32.8 (12.5)	31.9 (11.7)	33.2 (12.9)	−0.35	0.73
Mental health	0–100	45.9 (11.5)	46.2 (9.6)	45.8 (12.2)	0.12	0.91

^a^Fisher’s Exact Test.

### Physical and mental health

Physical health scores showed statistically significant improvements between baseline (*M* = 32.8, *SD* = 12.5) and two-year follow-up (*M* = 47.5, *SD* = 11.2, *t*(58) = 8.54, *p* < 0.001, *d* = 1.24). For the 50 participants who attended the 2-week follow-up immediately following the patient education course, there was also evidence of immediate improvement in physical health between baseline (*M* = 32.6, *SD* = 12.7) and 2-week follow-up (*M* = 35.0, *SD* = 12.1, *p* = 0.02).

Mental health scores, however, did not show statistically significant changes between baseline (*M* = 45.9, *SD* = 11.5) and two-year follow-up (*M* = 48.2, *SD* = 11.9, *t*(58) = 1.42, *p* = 0.16, *d* = 0.20). Similarly, the 50 participants who attended the 2-week follow-up showed no immediate change in mental health from baseline (*M* = 46.3, *SD* = 11.9) to after the course (*M* = 46.6, *SD* = 12.6, *p* = 0.87).

### Correlates of physical and mental health

The first set of multivariate analyses showed that higher self-esteem was associated with better mental health at baseline, controlling for work status and self-efficacy levels (Table 2). The regression model accounted for 34.8% of the variance in baseline mental health. None of the variables were associated with physical health at baseline.

**Table 2:** Bivariate relationships (Pearson’s *r*) and multivariate linear regression analysis (standardized beta coefficients) with SF-12 physical and mental health scores at baseline as dependent variables (*N* = 59)

Independent variables	Physical health at baseline			Mental health at baseline		
	*r*	*β*	*p*	*r*	*β*	*p*
Step 1. Socio-demographic and personal variables at baseline						
Work status (reference = working)	−0.13	−0.19	0.19	−0.25	−0.08	0.52
Self-esteem (RSES-4, high scores = low self-esteem)	0.07	0.22	0.18	−0.58**	−0.48	<0.01
Self-efficacy (GSE, high scores = high self-efficacy)	0.07	0.16	0.30	0.40**	0.13	0.31
Explained variance (R^**2**^ **)**		5.1%	0.41		34.8%	<0.001

**p < 0.01

The second set of multivariate analyses, controlling for work status, personal variables, surgery, and baseline levels of physical health, showed that having bariatric surgery during the first year after the patient education course was associated with better physical health at two-year follow-up (Table 3). The full model accounted for 42.5% of the variance in physical health. Except for the baseline mental health score, none of the variables were significantly associated with the mental health score at two-year follow-up. The full model explained 25.5% of the variance in mental health.

**Table 3:** Bivariate relationships (Pearson’s *r*) and multivariate linear regression analysis (standardized beta coefficients) using baseline variables and surgical treatment to predict SF-12 physical and mental health scores at two-year follow-up (*N* = 59)

Independent variables	Physical health at two-year follow-up			Mental health at two-year follow-up		
	*r*	*β*	*p*	*r*	*β*	*p*
Step 1. Socio-demographic and personal variables at baseline						
Work status (reference = working)	−0.32*	−0.33	0.007	−0.29*	−0.16	0.22
Self-esteem (RSES-4, high scores = low self-esteem)	0.18	0.27	0.05	−0.39**	−0.20	0.23
Self-efficacy (GSE, high scores = high self-efficacy)	−0.10	0.01	0.97	0.17	−0.65	0.52
**Explained variance (R** ^2^)		19.9%	0.01		18.2%	0.01
Step 2. Subsequent treatment						
Surgery (reference = no surgery)	0.41**	0.29	0.01	0.01	−0.09	0.52
Change in explained variance		4.9%	0.07		0.8%	0.47
Explained variance (R^2^)		24.7%			19.0%	
Step 3. Baseline quality of life (high scores = high quality)						
Baseline physical health	0.39**	0.37	0.002	−0.11	-	-
Baseline mental health	−0.13	-	-	0.45**	0.33	0.03
Change in explained variance		12.6%	0.002		7.2%	0.03
Explained variance (R^2^)		37.4%			26.3%	

*Note.* The displayed results from the multivariate analysis are controlled for all predictors in the model.

*p < 0.05

**p < 0.01

In the third set of multivariate analyses, baseline levels of self-esteem and self-efficacy were replaced with variables representing the change in self-esteem and self-efficacy during the two-year follow-up period (Table 4). Having had bariatric surgery was associated with better physical health at two-year follow-up, but changes in self-esteem and self-efficacy were not. The final model explained 40.8% of the variance in physical health. Controlling for the same variables, increases in self-efficacy and increases in self-esteem during the follow-up period were both associated with better mental health at two-year follow-up. The final model explained 51.8% of the variance in mental health.

**Table 4:** Bivariate relationships (Pearson’s *r*) and multivariate linear regression analysis (standardized beta coefficients) using change in personal variables to predict SF-12 physical and mental health scores at two-year follow-up (*N* = 59)

Independent variables	Physical health two-year follow-up			Mental health two-year follow-up		
	*r*	*β*	*p*	*r*	*β*	*p*
Step 1. Socio-demographic variables and change in personal variables						
Work status (reference = working)	−0.32*	−0.19	0.14	−0.29*	−0.16	0.18
Self-esteem change (RSES-4, high scores = decreased self-esteem)	−0.23	−0.15	0.36	−0.27*	−0.32	0.03
Self-efficacy change (GSE, high scores = increased self-efficacy)	0.11	−0.05	0.74	0.39**	0.34	0.02
**Explained variance (R** ^**2**^ **)**		18.9%	0.01		21.0%	<0.01
Step 2. Subsequent treatment						
Surgery (reference = no surgery)	0.41**	0.38	<0.01	0.01	−0.20	0.10
Change in explained variance		5.5%	0.07		2.4%	0.22
Explained variance (R^2^)		24.4%			23.4%	
Step 3. Baseline quality of life (high scores = high quality)						
Baseline physical health	0.39**	0.42	0.001	−0.11	-	-
Baseline mental health	−0.13	-	-	0.45**	0.58	0.001
Change in explained variance		16.4%	0.001		28.4%	<0.001
Explained variance (R^2^		40.8%			51.8%	

*Note*. The displayed results from the multivariate analysis are controlled for all predictors in the model.

*p < 0.05

**p < 0.01

## Discussion

The participants in our study had substantial improvements in their physical health in the 2 years following the patient education course, but had no statistically significant improvement in mental health. A similar two-year follow-up study of morbidly obese patients undergoing bariatric surgery also showed a stronger improvement in physical health than in mental health, even though their patients had considerably higher scores for both HRQoL components at baseline [14].

In our study, having better physical health at baseline and having gastric surgery during the two-year period were both significant predictors of physical health at two-year follow-up, controlling for work status and personal factors (Table 3). Having paid work at baseline was also related to better physical health at two-year follow-up, but the relationship did not reach our criterion for statistical significance, possibly due to the small number of people not working, which likely resulted in low statistical power. Unexpectedly, lower baseline self esteem was associated with better physical health at 2-year follow-up, although this relationship did not quite reach our criterion for clinical significance. When we evaluated changes in personal variables over the two-year study period (Table 4), we found that changes in self-esteem and self-efficacy were unrelated to physical health at the two-year follow-up, consistent with our hypothesis.

In terms of mental health, higher self-esteem at baseline was strongly related to mental health both at baseline (Table 2) and the two-year follow-up (Table 3), even after controlling for work status and level of self-efficacy. However, when mental health at baseline was included in the multiple regression analysis predicting mental health at two-year follow-up, the relationship between higher self-esteem and mental health at follow-up was attenuated and no longer statistically significant (Table 3). In fact, mental health at baseline was the only baseline predictor of mental health at two-year follow-up. However, when we evaluated changes in personal variables over the two-year study period, we found, consistent with our hypothesis, that improvements in self-esteem and self-efficacy were also associated with better mental health at the two-year follow-up, even controlling for surgery treatment and baseline mental health scores (Table 4).

At baseline, higher self-esteem was directly associated with better mental health (Table 2). Other studies have pointed to self-esteem as one of the important aspects of mental health [27–29]. Self-esteem encompasses a sense of self-worth and positive feelings about oneself, and such feelings may increase a person’s ability to cope with stress, demanding interpersonal relationships, and daily life challenges in general. Thus, the detected association between higher self-esteem and better mental health fits with previous research [27, 28]. A systematic review from 2015 [30] concluded that relatively few community-based obesity prevention trials have measured mental health, and that mental health measures ought to be included in more studies.

While self-esteem at baseline was associated with mental health at two-year follow-up in the bivariate analyses, neither baseline self-esteem, nor baseline self-efficacy was associated with mental health at two-year follow-up in the multivariate model (Table 3). The absence of an association between baseline self-esteem and mental health 2 years later when controlling for baseline mental health may indicate that personal factors, such as self-esteem, are not stable but changeable characteristics. In fact, the patient education courses for the obese participants were grounded in the belief that psychological change is one aspect of the lifestyle change process. Helping the person towards an improved sense of self and towards a more positive view of his or her ability to make lifestyle changes are explicit goals of the patient education courses for obese participants. Another explanation may be that controlling for mental health at baseline eliminates the association between baseline self-esteem and mental health at two-year follow-up, leaving little variance left to be uniquely explained by self-esteem.

The subsequent analysis, using self-esteem and self-efficacy change scores as independent variables in the model, found that positive changes in these personal factors were associated with better mental health at two-year follow-up (Table 4). These results indicate that both self-esteem and self-efficacy are important aspects of mental health [29, 31]. Further, the results suggest that changes in self-esteem and self-efficacy contribute substantially to an understanding of mental health from a long-term perspective. Thus, the impact of psychological change for later mental health status should not be disregarded.

This study showed that physical health was markedly improved in the participants over the two-year follow-up period, whereas mental health was not substantially changed. Personal factors contributed substantially to variations in mental health among obese persons with relatively large effect sizes [26, 32]. Socio-demographic variables were less important in explaining the variance in mental health than in physical health when we controlled for the participants level of self-esteem and self-efficacy at baseline. Our findings also suggest that the factors related to the physical health of obese persons are different from those related to their mental health. Further, they indicate that addressing personal factors, such as self-esteem and self-efficacy, in intervention studies might contribute to improved mental health in this population.

### Strengths and limitations

The study had an acceptable response rate of 77% at baseline, but a rather high attrition rate of 58% at the two-year follow-up. However, a comparison of the socio-demographic characteristics of the study sample and the non-participants revealed no differences in relation to age or gender. Similarly, attrition analyses showed that participants who dropped out at two-year follow-up did not differ from the study sample with regard to age, gender, baseline personal factors, or baseline physical or mental health. Thus, despite the high attrition, the findings reported in this study are likely to be relatively representative of people with morbid obesity attending patient educational course while on a waiting list for treatment. However, individuals in the present sample might not be representative of all morbidly obese persons. Those who are on the waiting list for treatment for their morbid obesity may be a self-selected sample, particularly troubled by their weight, or especially susceptible to developing problems that may result from their excess weight. Furthermore, significant differences between community and clinical samples of severely obese individuals have been reported [33, 34], and thus, the findings in our clinical sample may not generalize to community populations.

Other strengths of the study were that we used standardized and validated instruments and that the participants responded by self-report questionnaires, which have been found to be less biased toward socially desirable responses than other modes, such as face-to-face interviews [35]. Since we recruited participants from the health promotional context of learning centres, we avoided asking them about their experiences of negative symptoms, concomitant diseases and weight at the first data collection in order to avoid interfering with the educational program. However, such factors may be mediators and/or modifiers of the relationship between personal factors and the patients’ physical and mental health [7, 8]. A recent study showed that physical and mental illnesses were factors related to the mental HRQoL in persons with obesity [7]. The relationship between self-esteem and mental health might well be confounded by depression. A study examining relationships of weight status to body image and depression in youth found higher depression scores and lower self-efficacy scores among obese persons [36]. Further, the 4-item version of RSES has been used in a small number of studies, and no cut off for low or high self-esteem has been established, which makes comparisons difficult. Finally, since we used a generic HRQoL measure, we can not be certain of how use of an obesity-specific instrument may have altered the results, or whether a disease-specific HRQoL may have been more sensitive to detecting changes in mental health.

### Implication for future studies of behavioural change and for patient education

As we have stated previously [7], there is a need to explore whether and to what degree an educational program can contribute to improved self-efficacy and self-esteem, and thereby increased quality of life among morbidly obese persons seeking treatment. If self-esteem and self-efficacy can be improved through an educational program, changes in these self-beliefs may help explain any observed improvements in HRQoL following the program [37]. The current study results suggest that providers of educational courses designed to prepare obese persons for medical and surgical treatment, as well as for lifestyle changes, should pay attention to low HRQoL and take into consideration the body and mind factors of the course participants. Moreover, these findings warrant further studies to investigate whether extra support provided before and/or after surgery to obese individuals with poor mental health can improve outcomes.

## Conclusions

Our study showed that people with morbid obesity on a waiting list for bariatric surgery improved their physical health during the 2 years after attending a tailored educational course. Improving self-esteem and self-efficacy may be important personal factors for maintaining mental health during this period.
